# Role of PI3K/AKT/mTOR signaling pathway and sirtuin genes in chronic obstructive pulmonary disease development

**DOI:** 10.18699/VJGB-23-62

**Published:** 2023-09

**Authors:** G.F. Korytina, L.Z. Akhmadishina, V.A. Markelov, Y.G. Aznabaeva, O.V. Kochetova, T.R. Nasibullin, A.P. Larkina, N.N. Khusnutdinova, N.Sh. Zagidullin, T.V. Victorova

**Affiliations:** Institute of Biochemistry and Genetics – Subdivision of the Ufa Federal Research Centre of the Russian Academy of Sciences, Ufa, Russia Bashkir State Medical University, Ufa, Russia; Institute of Biochemistry and Genetics – Subdivision of the Ufa Federal Research Centre of the Russian Academy of Sciences, Ufa, Russia; Institute of Biochemistry and Genetics – Subdivision of the Ufa Federal Research Centre of the Russian Academy of Sciences, Ufa, Russia Bashkir State Medical University, Ufa, Russia; Bashkir State Medical University, Ufa, Russia; Institute of Biochemistry and Genetics – Subdivision of the Ufa Federal Research Centre of the Russian Academy of Sciences, Ufa, Russia; Institute of Biochemistry and Genetics – Subdivision of the Ufa Federal Research Centre of the Russian Academy of Sciences, Ufa, Russia; Institute of Biochemistry and Genetics – Subdivision of the Ufa Federal Research Centre of the Russian Academy of Sciences, Ufa, Russia; Institute of Biochemistry and Genetics – Subdivision of the Ufa Federal Research Centre of the Russian Academy of Sciences, Ufa, Russia; Bashkir State Medical University, Ufa, Russia; Bashkir State Medical University, Ufa, Russia

**Keywords:** chronic obstructive pulmonary disease, PI3K/AKT/mTOR signaling pathway, sirtuins, cellular senescence, oxidative stress, хроническая обструктивная болезнь легких, PI3K/AKT/mTOR-сигнальный каскад, сиртуины, клеточное старение, окислительный стресс

## Abstract

Chronic obstructive pulmonary disease (COPD) is a multifactorial disease of the respiratory system which develops as a result of a complex interaction of genetic and environmental factors closely related to lifestyle. We aimed to assess the combined effect of the PI3K/AKT/mTOR signaling pathway (PIK3R1, AKT1, MTOR, PTEN) and sirtuin (SIRT1, SIRT3, SIRT6) genes to COPD risk. SNPs of SIRT1 (rs3758391, rs3818292), SIRT3 (rs3782116, rs536715), SIRT6 (rs107251), AKT1 (rs2494732), PIK3R1 (rs10515070, rs831125, rs3730089), MTOR (rs2295080, rs2536), PTEN (rs701848, rs2735343) genes were genotyped by real-time polymerase chain reaction (PCR) among 1245 case and control samples. Logistic regression was used to detect the association of SNPs in different models. Linear regression analyses were performed to estimate the relationship between SNPs and lung function parameters and smoking pack-years. Significant associations with COPD were identified for SIRT1 (rs3818292) (P = 0.001, OR = 1.51 for AG), SIRT3 (rs3782116) (P = 0.0055, OR = 0.69) and SIRT3 (rs536715) (P = 0.00001, OR = 0.50) under the dominant model, SIRT6 (rs107251) (P = 0.00001, OR = 0.55 for СT), PIK3R1: (rs10515070 (P = 0.0023, OR = 1.47 for AT), rs831125 (P = 0.00001, OR = 2.28 for AG), rs3730089 (P = 0.0007, OR = 1.73 for GG)), PTEN: (rs701848 (P = 0.0015, OR = 1.35 under the log-additive model), and rs2735343 (P = 0.0001, OR = 1.64 for GC)). A significant genotype-dependent variation of lung function parameters was observed for SIRT1 (rs3818292), SIRT3 (rs3782116), PIK3R1 (rs3730089), and MTOR (rs2536). Gene-gene combinations that remained significantly associated with COPD were obtained; the highest risk of COPD was conferred by a combination of G allele of the PIK3R1 (rs831125) gene and GG of SIRT3 (rs536715) (OR = 3.45). The obtained results of polygenic analysis indicate the interaction of genes encoding sirtuins SIRT3, SIRT2, SIRT6 and PI3KR1, PTEN, MTOR and confirm the functional relationship between sirtuins and the PI3K/AKT/mTOR signaling pathway.

## Abstract (ru)

Хроническая обструктивная болезнь легких (ХОБЛ) – многофакторное заболевание дыхательной системы, развивающееся в результате комплексного взаимодействия молекулярно-генетических и средовых факторов, тесно связанных с образом жизни. Цель исследования – анализ комбинированного вклада генов PI3K/AKT/mTOR-сигнального каскада (PIK3R1, AKT1, MTOR, PTEN) и сиртуинов (SIRT1, SIRT3, SIRT6) в риск развития хронической обструктивной болезни легких. В работе использованы образцы ДНК 1245 индивидов. Полиморфные варианты генов SIRT1 (rs3758391, rs3818292), SIRT3 (rs3782116, rs536715), SIRT6 (rs107251), AKT1 (rs2494732), PIK3R1 (rs10515070, rs831125, rs3730089), MTOR (rs2295080, rs2536), PTEN (rs701848, rs2735343) анализировали методом полимеразной цепной реакции в реальном времени. Логистическую регрессию использовали для выявления ассоциации полиморфных локусов в различных моделях. Проводили линейный регрессионный анализ для оценки вклада генотипов изучаемых локусов в вариабельность показателей функции внешнего дыхания и индекса курения. Установлена ассоциация генов: SIRT1 (rs3818292) (P = 0.001, OR = 1.51 для генотипа AG), SIRT3 (rs3782116) (P = 0.0055, OR = 0.69) и SIRT3 (rs536715) (P = 0.00001, OR = 0.50) в доминантной модели; SIRT6 (rs107251) (P = 0.00001, OR = 0.55 для генотипа СT), PIK3R1: (rs10515070 (P = 0.0023, OR = 1.47 для генотипа AT), rs831125 (P = 0.00001, OR = 2.28 для генотипа AG), rs3730089 (P = 0.0007, OR = 1.73 для генотипа GG)) и PTEN: (rs701848 (P = 0.0015, OR = 1.35 в лог-аддитивной модели) и rs2735343 (P = 0.0001, OR = 1.64 для генотипа GC)). Обнаружена вариабельность показателей функции легочного дыхания в зависимости от полиморфных вариантов генов SIRT1 (rs3818292), SIRT3 (rs3782116), PIK3R1 (rs3730089) и MTOR (rs2536). Идентифицированы ген-генные сочетания, ассоциированные с ХОБЛ; наибольший риск развития ХОБЛ определялся сочетанием аллеля G гена PIK3R1 (rs831125) с генотипом GG гена SIRT3 (rs536715) (OR = 3.45). Полученные результаты полигенного анализа указывают на взаимодействие генов, кодирующих сиртуины SIRT3, SIRT2, SIRT6 и PI3KR1, PTEN, MTOR, и находят подтверждение в функциональной взаимосвязи сиртуинов и PI3K/AKT/mTOR-сигнального каскада.

## Introduction

Chronic obstructive pulmonary disease (COPD) is a multifactorial respiratory system disease that affects the distal parts of the respiratory tract (bronchi, bronchioles) and lung parenchyma with lung emphysema development (Chuchalin et al., 2022). Сhronic obstructive pulmonary disease develops as a result of complex interaction between molecular genetic and environmental factors, closely related to lifestyle, and smoking is considered the main cause of COPD (Ragland et al., 2019). Published data suggest that the COPD pathogenesis may involve dysregulation of stress responses that inhibit cellular senescence, which includes a wide range of signaling cascades and their regulators (Ryter et al., 2018).

The PI3K/AKT/mTOR intracellular signaling pathway is a universal pathway controlling cell growth, metabolism, and proliferation (Ersahin et al., 2015). The key components of this signaling pathway are phosphatidylinositol-3 kinase (PI3K), serine/threonine protein kinase (AKT), and serine/threonine kinase (mammalian target of rapamycin, mTOR) (Ersahin et al., 2015). Signal transduction through the PI3K/AKT/ mTOR signaling cascade is essential for cellular senescence. This signaling pathway is inhibited by the tyrosine phosphatases PTEN (phosphatase and tensin homolog) and SHIP-1 (inositol polyphosphate-5-phosphatase D). Both enzymes have oxidation-sensitive cysteine residue in the active region (Worby, Dixon, 2014).

Oxidative stress is the main mechanism that causes accelerated senescence through its damaging effects on DNA and the PI3K/AKT/mTOR signaling pathway activation (Wang et al., 2013). In COPD and other age-associated diseases, the expression of genes encoding endogenous antioxidant molecules is reduced, which leads to an increased level of oxidative stress and cellular senescence activation (Kirkham, Barnes, 2013). NAD-dependent protein deacetylases from the sirtuins family are considered as potential factors that decrease senescence (Ito, Barnes, 2009).

We aimed to assess the combined effect of the PI3K/AKT/ mTOR signaling pathway (PIK3R1, AKT1, MTOR, PTEN) and sirtuins (SIRT1, SIRT3, SIRT6) genes on COPD risk.

## Materials and methods

DNA samples were collected from unrelated subjects who were Tatars in ethnicity and resided in the Republic of Bashkortostan. The study was approved by the Ethics Committee at the Institute of Biochemistry and Genetics (Protocol No 17, December 7, 2010). All participants of this study provided written informed consent. The COPD group included 621 patients (539 (86.79 %) males and 82 (13.21 %) females) with a mean age of 64.42 ± 10.71 years. There were 510 (82.13 %) smokers and former smokers and 111 (17.87 %) nonsmokers in the COPD group. The smoking index was 45.34 ± 23.84 pack years in the smokers and former smokers. The control group included 624 subjects (555 (88.94 %) males and 69 (11.06 %) females) with a mean age of 59.67 ± 12.31. There were 526 (84.29 %) smokers and former smokers and 98 (15.71 %) nonsmokers in the group; the smoking index was 38.75 ± 24.87 pack years in the smokers

In all patients, spirometry was performed to assess lung function, including vital capacity (VC), forced vital capacity (FVC), forced expiration volume in the first second (FEV1), and the FEV1/FVC ratio. The group of patients had the following parameters (% of normal levels): FEV1 = 40.75 ± 18.33; FVC = 45.01 ± 18.22; VC = 49.32 ± 14.34; FEV1/FVC = 59.5 ± 12.34. Inclusion and exclusion criteria for the COPD and control have been previously described (Korytina et al., 2019).

Genotyping. DNA was isolated from peripheral blood leukocytes by phenol-chloroform extraction. The set included SNPs of the following genes: SIRT1 (rs3758391, rs3818292), SIRT3 (rs3782116, rs536715), SIRT6 (rs107251), AKT1 (rs2494732), PIK3R1 (rs10515070, rs831125, rs3730089), MTOR (rs2295080, rs2536), PTEN (rs701848, rs2735343) (Supplementary Material 1)1.

Supplementary Materials are available in the online version of the paper: http://vavilov.elpub.ru/jour/manager/files/Suppl_Korytina_Engl_27_5.pdf


The SNPs were selected for the study based on the following criteria: functional effect of SNP on gene expression or relation to non-synonymous substitutions, and/or associations with complex human diseases; minor allele frequency (MAF) of ≥ 5 % in the European populations according to the National Center for Biotechnology Information database (http://www.ncbi.nlm.nih.gov/projects/SNP/). The functional significance of the SNPs was verified using RegulomeDB Version 1.1 (https://regulomedb.org), SNPinfo Web Server (https://snpinfo.niehs.nih.gov), and HaploReg v3 (Ward, Kellis, 2016). Data were presented in Supplementary Material 2. SNP genotyping was performed by real-time polymerase chain reaction (PCR) using commercial kits for fluorescence detection (https://www.oligos.ru, DNK-Sintez, Russia) and a BioRad CFX96TM instrument (Bio-Rad Laboratories, United States). The methods of analysis were described in detail previously (Korytina et al., 2019).

Statistical analyses. Statistical analyses of the results were performed using the software packages IBM SPSS Statis- tics 22.0 and PLINK v. 1.07 (Purcell et al., 2007). The methods were described in detail previously (Korytina et al., 2019). Association analyses of allele or genotype combinations with COPD were carried out using the APSampler 3.6.1 program (http://sourceforge.net/projects/apsampler/). The Benjiamini–Hochberg correction for multiple testing was performed using special software (http://www.sdmproject. com/utilinies/?show=FDR) to decrease the false discovery rate (FDR) and to obtain Рcor- FDR. The linkage disequilibrium structure LD (D’) and haplotype frequencies were calculated with Haploview 4.2.

## Results

Before analyzing the association of candidate gene alleles with COPD, we verified whether the genotype frequency distributions corresponded to the Hardy–Weinberg equilibrium and evaluated minor allele frequencies (MAF) both in the combined group of patients and healthy subjects and in either group individually (see Supplementary Material 1). All studied SNPs were in Hardy–Weinberg equilibrium in the control group: SIRT1 (rs3758391) (PX-B = 0.24), SIRT1 (rs3818292) (PX-B = 0.47), SIRT3 (rs3782116) (PX-B = 0.5), SIRT3 (rs536715) (PX-B = 0.75), SIRT6 (rs107251) (PX-B = 0.67), AKT1 (rs2494732) (PX-B = 0.2), PIK3R1 (rs10515070) (PX-B = 0.65), PIK3R1 (rs831125) (PX-B = 0.25), PIK3R1 (rs3730089) (PX-B = 0.22), MTOR (rs2295080) (PX-B = 0.15), MTOR (rs2536) (PX-B = 0.24), PTEN (rs701848) (PX-B = 0.85), PTEN (rs2735343) (PX-B = 0.06).

The groups of COPD patients and healthy controls differed significantly in the genotypes and/or alleles frequency distributions of SIRT1 (rs3818292), SIRT3 (rs3782116, rs536715), SIRT6 (rs107251), AKT1 (rs2494732), PIK3R1 (rs10515070, rs831125, rs3730089), and PTEN (rs701848, rs2735343) (Table 1). Statistically significant results of association analysis of the studied gene loci and COPD are shown in Table 2.

**Table 1.**
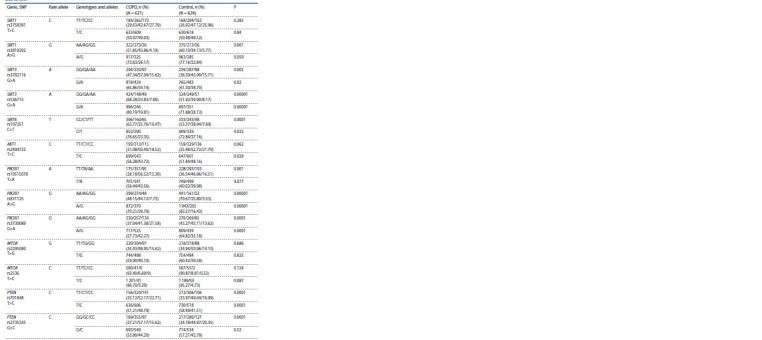
Genotypes and alleles distribution of the studied PI3K/AKT/mTOR signaling pathway and sirtuins genes loci in COPD and control P is the significance of group differences in allele and genotype frequencies (sample χ2 homogeneity test).

**Table 2.**
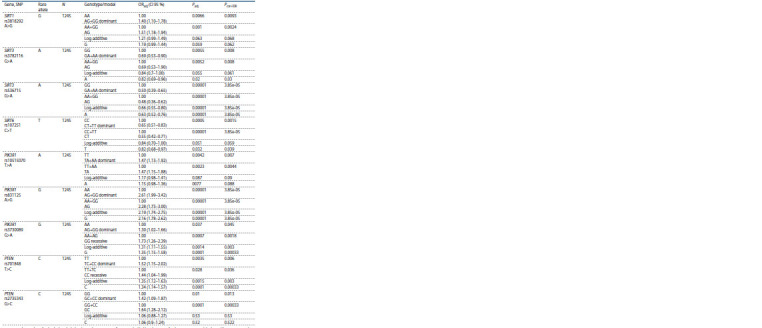
Significant association of the studied PI3K/AKT/mTOR signaling pathway and sirtuins genes loci with COPD N is the number of individuals included in the analysis; Padj – significance in the likelihood ratio test for the regression model adjusted for age, sex, smoking status and pack-years; ORadj – adjusted odds ratio and CI 95 % – confidence interval; Pcor-FDR – significance after the FDR correction; in the log-additive model per rare allele dosage, the rare allele dosage increases in the following order: homozygote for the common allele (0) – heterozygote, (1) – homozygote for the rare allele (2).

An association of SIRT1 (rs3818292) with COPD was established in the dominant model (Padj = 0.0066, OR = 1.40). The risk of COPD was increased in heterozygous individuals (Padj = 0.001, OR = 1.51). An association of COPD with the heterozygous genotype (Padj = 0.0052, OR = 0.69) and in the dominant model of SIRT3 (rs3782116) and in the dominant (Padj = 0.00001, OR = 0.50), log-additive (Padj = 0.00001, OR = 0.66) models and with the heterozygous genotype (Padj = 0.00001, OR = 0.48) of SIRT3 (rs536715). It should be noted that in both cases the COPD risk was associated with the frequent G allele (rs3782116 – OR = 1.21 95 % CI 1.03–1.43 и rs536715 – OR = 1.58 95 % CI 1.32–1.91) and the GG genotype (rs3782116 – OR = 1.44 95 % CI 1.16–1.81 и rs536715 – OR = 1.99 95 % CI 1.58–2.51).

We carried out a linkage disequilibrium analysis of the rs3758391 and rs3818292 loci of the SIRT1, rs3782116 and rs536715 of the SIRT3, which showed the absence of linkage disequilibrium between the loci of the SIRT1 gene (D′ = 0.168, r2 = 0.097) and the SIRT3 gene (D′ = 0.28, r2 = 0.011). Based on the obtained data, haplotypes association analysis was not performed. Association of SIRT6 (rs107251) with the development of COPD was detected in the dominant model (Padj = 0.0005, OR = 0.65), but the association with the heterozygous CT genotype was more significant (Padj = 0.00001, OR = 0.55). The risk of COPD was associated with the CC ge- notype of SIRT6 (rs107251) (OR = 1.54 95 % CI 1.23–1.93).

We have identified the association SNPs of the PIK3R1 gene (rs10515070, rs831125, rs3730089) with COPD. The risk of COPD was associated with heterozygous genotypes of the studied SNPs of the PIK3R1 gene: rs10515070 (Padj = 0.0023, OR = 1.47), rs831125 (Padj = 0.00001, OR = 2.28) and with the GG genotype of rs3730089 (Padj = 0.0007, OR = 1.73). We showed the absence of linkage disequilibrium between rs10515070, rs831125, rs3730089 of the PIK3R1 gene: for rs10515070 and rs831125 (D′ = 0.02, r2 = 0.00), for rs10515070 and rs3730089 (D′ = 0.127, r2 = 0.008), for rs831125 and rs3730089 (D′ = 0.155, r2 = 0.005).

Based on the obtained data, haplotypes association analysis was not performed. Association of PTEN (rs701848) and COPD was established in the dominant (Padj = 0.0035, OR = 1.52), recessive (Padj = 0.028, OR = 1.44) and log-additive models Padj = 0.0015, OR = 1.35). We have identified the association of PTEN (rs2735343) with COPD in the log-dominant model (Padj = 0.01, OR = 1.42) and for the heterozygous genotype (Padj = 0.0001, OR = 1.64). Linkage disequilibrium between the rs701848 and rs2735343 was not detected (D′ = 0.234, r2 = 0.035), thus, haplotype association analysis was not performed.

**Association of the studied genes loci and quantitative phenotypes with lung function parameters and smoking index**

Lung function decline is a key clinical feature of airway obstruction in COPD that indicates progression of the disease. We investigated the relationship between the studied genes loci and lung function parameters: Forced Vital Capacity (FVC), Forced Expiration Volume in 1 s (FEV1), and FEV1/FVC ratio in COPD patients (Table 3). The heterozygous genotype of PIK3R1 (rs3730089) (P = 0.013) and the TT genotype of MTOR (rs2536) (P = 0.013) were associated with a decrease in the FVC value. Carriers of the SIRT3 (rs3782116) AA genotype exhibited a higher FVC value (P = 0.0015).

**Table 3.**
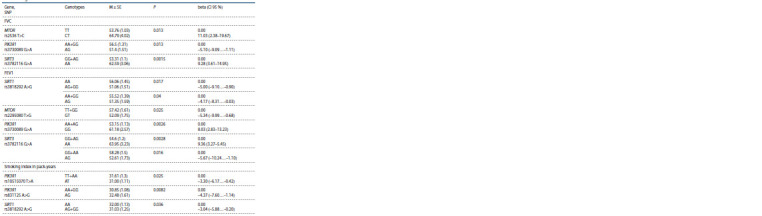
Association of the PI3K/AKT/mTOR signaling pathway and sirtuins gene loci with lung function parameters and smoking index M ± SE is the mean ± standard error of the mean; P is the significance level for the regression equation; beta (95 % CI) is the regression coefficient (95 % confidence interval of the coefficient).

Individuals that presented the AA genotype of SIRT1 (rs3818292) (P = 0.017), the GG genotype of PIK3R1 (rs3730089) (P = 0.025), and the AA genotype of SIRT3 (rs3782116) (P = 0.0028) showed a significant increase in their FVC. Meanwhile, carriers of the heterozygous genotypes of SIRT1 (rs3818292) (P = 0.04), MTOR (rs2295080) (P = 0.025), and SIRT3 (rs3782116) (P = 0.016) exhibited a lower FVC value (see Table 3). The carriers of the heterozygous genotype of PIK3R1 (rs831125) (P = 0.0082), the AA genotype of SIRT1 (rs3818292) (P = 0.036) had a significantly higher smoking index.

**Analysis of gene-gene interactions**

Using the APSampler algorithm, we have identified gene-gene combinations significantly associated with сhronic obstructive pulmonary disease. In order to identify significant interactions of functionally related sirtuins genes, SIRT2 (rs10410544) was included in the analysis (Korytina et al., 2019). We obtained 2324 patterns associated with сhronic obstructive pulmonary disease. Table 4 shows the results of the most significant genegene combinations with PFDR less than 0.05 and OR more than 2.0 for risk combinations) or less than 0.35 for protective combinations. A total of 19 gene-gene combinations fulfilled the above-mentioned criteria. Nine patterns were associated with an increased risk of COPD; ten were protective. Allele G and genotype GG of PIK3R1 (rs831125) contributed to the most significant combinations associated with COPD risk (four patters).

**Table 4.**
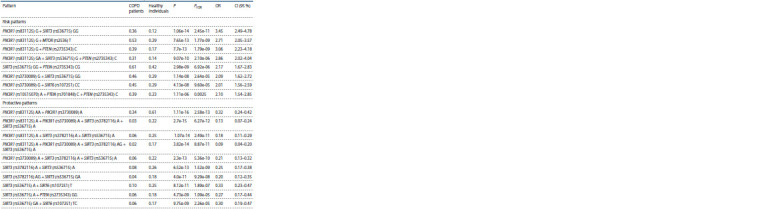
Gene-gene combinations of the PI3K/AKT/mTOR signaling pathway and sirtuins gene loci associated with COPD P-value is the significance level for Fisher's test, PFDR – is the FDR value after FDR correction; OR is odds ratio, 95 % CI is the 95 % confidence interval for the OR.

The highest risk of COPD was conferred by the combination of these variants of PIK3R1 (rs831125) with the GG genotype of SIRT3 (rs536715) (OR = 3.45); and with the C allele of PTEN (rs2735343) (OR = 3.06) and their combination: genotype GA of PIK3R1 (rs831125) together with the G allele of SIRT3 (rs536715) and the C allele of PTEN (rs2735343) (OR = 2.86). The analysis on the gene-gene interaction of the studied gene loci established an association of the T allele of MTOR (rs2536) only in combinations with the G allele of PIK3R1 (rs831125) (OR = 2.71). Three of the identified patterns included the G allele of PIK3R1 (rs3730089) in combination with the GG genotype of SIRT3 (rs536715) (OR = 2.09), or with the CC genotype of SIRT6 (rs107251) (OR = 2.01).

The most significant protective patterns included the A allele or the AA genotype of PIK3R1 (rs831125) and the A allele of PIK3R1 (rs3730089) in combination with the A allele or the AG genotype of SIRT3 (rs3782116) and the A allele of SIRT3 (rs536715) (see Table 4). Thus, the PIK3R1 (rs831125), PIK3R1 (rs3730089), and SIRT3 (rs536715) loci exhibited an allele-specific effect, when the G allele of PIK3R1 (rs831125), the G allele of PIK3R1 (rs3730089), and the G allele and the GG genotype of SIRT3 (rs536715) were part of risk patterns, and alternative alleles of the same polymorphic loci were present in protective patterns.

## Discussion

As a result our study, significant associations between the SIRT1 (rs3818292), SIRT3 (rs3782116, rs536715), SIRT6 (rs107251) polymorphic variants and COPD were found. SIRT1 is the most studied member of the mammalian sirtuin family. It has been shown that SIRT1 plays an important role in signaling pathways involved in cellular senescence and cell death (Finkel et al., 2009). SIRT1 deacetylates many key regulatory proteins and transcription factors involved in DNA repair, inflammation, expression of antioxidant genes, and cellular senescence, including the PI3K/AKT/mTOR signaling pathway genes, transcription factor FOXO3a, p21, p16, Klotho proteins (Cao et al., 2013).

Increased expression of SIRT1 inhibits the TGF-β1/SMAD3 signaling pathway and impairs epithelial-mesenchymal transformation, which leads to a decrease in COPD-associated airway remodeling (Zhang et al., 2022). SIRT1 levels are reduced in peripheral pulmonary and peripheral blood mononuclear cells of patients with COPD (Rajendrasozhan et al., 2008).

We found that the COPD risk is higher in heterozygous carriers of SIRT1 (rs3818292). Moreover, this polymorphic variant demonstrates an association with a decrease in FVС1, which reflects the progression of the disease. Functional analysis showed that SIRT1 (rs3818292) is in linkage with a SNP in the 5′-untranslated DNA region (rs3740051) that changes the NFKB transcription factor binding site. We did not associate SIRT1 (rs3758391) with COPD. The results of our study are in agreement with the previously published data reported for the Han Chinese population (Gao et al., 2018). According to functional analysis, rs3758391 is located in the promoter region of the SIRT1 gene, and the C variant disrupts binding sites for several transcription factors and regulatory proteins, affecting gene expression. The role of rs3758391 in the development of age-associated diseases is well known (Wu et al., 2022).

Mitochondrial dysfunction in respiratory epithelial cells plays an important role in the pathogenesis of COPD (Zhang et al., 2022). SIRT3 is the main mitochondrial deacetylase regulating many enzymes involved in energy metabolism, respiratory chain components, the tricarboxylic acid cycle, ketogenesis, and fatty acid beta-oxidation (Wu et al., 2022).

SIRT3 can directly control the production of reactive oxygen species (ROS) by deacetylating manganese superoxide dismutase (SOD2), the main mitochondrial antioxidant enzyme (Dikalova et al., 2017). SIRT3 is involved in regulating the activity of the DNA repair enzyme OGG1, which leads to increased damage to mtDNA and apoptosis of alveolar epithelial cells (Sun et al., 2018). A number of studies have shown the SIRT3 association with various complex diseases (Wu et al., 2022).

We studied the association between two SIRT3 gene functional polymorphisms (rs3782116 and rs536715) and сhronic obstructive pulmonary disease. Functional analysis showed that rs3782116 is located in the region of binding sites for hsa-miR-328; polymorphic loci rs3782116 and rs536715 are located in DNA regions that bind regulatory proteins. Both polymorphic loci were associated with COPD in our cohort; the disease development risk was associated with the G alleles of rs3782116 and rs536715. It should be noted that the carriers of the homozygous rare allele A of rs3782116 of the SIRT3 gene had higher values of VC and FVC1. The contribution of SIRT3 variants to COPD has not been studied previously. At the same time, effects of the SIRT3 gene SNPs have been fairly extensively investigated in age-associated diseases in which oxidative stress and cellular senescence play a key role (Song et al., 2022).

We obtained significant associations of SIRT6 (rs107251) with COPD; the frequent C allele is associated with COPD risk, while the heterozygous genotype has a protective effect on the disease development. rs107251 is located in the DNA region that binds to the SOX8 regulatory protein and it is in close linkage with rs350846, localized in the 3-non-translational region of the SIRT6 gene – a binding site for several miRNAs (hsa-miR-1207-5p, hsa-miR-24, hsa-miR-34a, hsamiR- 644, hsa-miR-940). SIRT6 participates in the regulation of genome stability, NF-kB signaling, glucose homeostasis, exhibits ADP-ribosyltransferase and histone deacetylase activity, plays a role in DNA repair and maintenance of telomeric chromatin integrity (Kugel, Mostoslavsky, 2014). In the study by N. Takasaka et al. (2014), a decrease in SIRT6 levels was shown in respiratory epithelial cells of COPD patients due to cigarette smoke exposure, leading to cellular senescence and disruption of autophagy processes. Association of the SIRT6 gene SNPs with COPD has not been studied previously, but there is evidence of their association with cardiovascular diseases, which are often comorbid pathologies in COPD (Song et al., 2022).

The PIK3R1 gene encodes regulatory subunit 1 of phosphoinositide 3-kinase, a key element of the PI3K/AKT/mTOR signaling cascade (Ersahin et al., 2015). We investigated three PIK3R1 gene polymorphic loci (rs10515070, rs831125, and rs3730089), which showed a significant association with COPD in our studied group. Carriers of rare alleles of these polymorphic loci had a high risk of COPD. In addition, we investigated the relationship between rs3730089 genotypes and VC and FVC1 values; thus, heterozygotes have lower values, and these results are in agreement with association analysis. Functional analysis showed that an intronic variant rs831125 is located in a binding site for regulatory proteins; rs3730089 is a missense variant with a “benign” effect according to the PolyPhen-2 database (http://genetics.bwh.harvard. edu/pph2/), located in a binding site for regulatory proteins and affecting splice sites. SNPs of the PIK3R1 gene have not been evaluated for COPD before. Previously, an association has been observed between rs3730089 and type 2 diabetes (Karadoğan et al., 2018).

The phosphatase PTEN regulates the activity of phosphoinositide- 3 kinase (PI3K) (Worby Dixon, 2014). Smoking as a major risk factor for COPD provokes oxidative stress, which, in turn, affects PTEN expression (Cai et al., 2022). We investigated two PTEN gene functional polymorphic loci; rs70184 is located in PTEN gene 3′ region and changes binding sites for hsa-miR-1252 and hsa-miR-1304 miRNA; rs2735343, located in the intronic region, affects binding sites for several regulatory proteins.

Significant associations with COPD in our sample were found with PTEN gene loci; thus, homozygous carriers of the rare C allele of rs701848 and heterozygous carriers of rs2735343 had a significant risk of COPD development.

Published data have demonstrated an association between PTEN (rs701848) and COPD; the risk was significantly reduced for homozygous T allele carriers, which is consistent with the data obtained for our sample (Hosgood et al., 2009). PTEN participates in the regulation of various biological processes, including cell proliferation, apoptosis, inflammatory reactions, transcription, and genomic stability (Cai et al., 2022). Decreased levels of PTEN lead to activation of PI3K signaling and increased cell senescence in COPD (Barnes et al., 2019). It has been shown that decreased PTEN activity in COPD increases the activity of matrix metalloprotease MMP9 in bronchial epithelial cells, which consequently contributes to the progression of inflammation and extracellular matrix degradation (Vannitamby et al., 2017).

The analysis of gene-gene interactions revealed significant synergy between polymorphic loci of genes encoding phosphoinositide-3-kinase (PIK3R1) and mitochondrial deacetylase (SIRT3), which were present in most significant combinations associated with an increased risk of сhronic obstructive pulmonary disease. The C allele in the PTEN (rs2735343) was part of four informative combinations associated with a high risk of сhronic obstructive pulmonary disease.

The results of polygenic analysis indicate the interaction of genes encoding sirtuins SIRT3, SIRT2, SIRT6 and PI3KR1, PTEN, MTOR and confirm the functional relationship between sirtuins and the PI3K/AKT/mTOR signaling pathway.

## Conclusion

The obtained results of single locus and polygenic analysis indicate the contribution of SIRT3 (rs3782116, rs536715), SIRT6 (rs107251) and PIK3R1 (rs10515070, rs831125, rs3730089) polymorphisms to COPD and interaction of genes enco- ding the key components of the PI3K/AKT/mTOR signalling pathway and sirtuins, and confirm the involvement of cellular senescence mechanisms with COPD development

## Conflict of interest

The authors declare no conflict of interest.
